# Machine Perfusion and Mitigating Risk Factors for Delayed Graft Function in Pediatric Kidney Transplant Recipients

**DOI:** 10.1111/petr.70429

**Published:** 2026-08-10

**Authors:** Kyle A. Merrill, Cassie Kirby, Teresa Ambrosino, Charles D. Varnell, Alexander Bondoc, Lakhmir Chawla, Stuart L. Goldstein, David K. Hooper

**Affiliations:** ^1^ Division of Nephrology, Dialysis and Transplantation University of Iowa Stead Family Children's Hospital Iowa City Iowa USA; ^2^ Stead Family Department of Pediatrics University of Iowa Carver College of Medicine Iowa City Iowa USA; ^3^ Center for Acute Care Nephrology Cincinnati Children's Hospital Medical Center Cincinnati Ohio USA; ^4^ Transplant Administration Cincinnati Children's Hospital Medical Center Cincinnati Ohio USA; ^5^ Division of Nephrology and Hypertension Cincinnati Children's Hospital Medical Center Cincinnati Ohio USA; ^6^ Department of Pediatrics University of Cincinnati College of Medicine Cincinnati Ohio USA; ^7^ James M. Anderson Center for Health Systems Excellence Cincinnati Children's Hospital Medical Center Cincinnati Ohio USA; ^8^ Division of Pediatric General and Thoracic Surgery Cincinnati Children's Hospital Medical Center Cincinnati Ohio USA; ^9^ Department of Surgery University of Cincinnati College of Medicine Cincinnati Ohio USA; ^10^ Veterans Affairs San Diego Healthcare System San Diego California USA

**Keywords:** delayed graft function, machine perfusion, pediatric kidney transplant

## Abstract

**Background:**

Kidney transplantation confers prolonged and improved quality of life versus dialysis for patients with end‐stage kidney disease (ESKD). Delayed graft function (DGF) increases the risk of allograft rejection and decreases allograft survival. In adults, prolonged warm (WIT) and cold ischemia time (CIT) increase risk of DGF, and machine perfusion (MP) during organ transportation reduces this risk. However, there is a paucity of data regarding risk factors for DGF in children.

**Methods:**

We retrospectively evaluated risk and protective factors for DGF in patients aged 18 months to 21 years who underwent kidney transplantation at Cincinnati Children's Hospital Medical Center between 11/1/2017 and 11/30/2021. DGF was defined as receiving dialysis within 7 days post‐transplant.

**Results:**

Nine of 89 (10%) patients developed DGF. Patients with DGF had longer median WIT (49 IQR: 44–57 vs. 37 min IQR: 30–43; *p* = 0.002) and were more likely to have received a deceased donor (DD) allograft (*p* = 0.04, RR1.7 (95% CI: 1.2–2.4)). There was no difference in CIT between those who did and did not experience DGF (464 IQR:329–788 vs. 271 min. IQR:271–788; *p* = 0.16). No patients who received DD‐MP kidneys developed DGF. DGF in recipients of DD‐non‐MP kidneys had longer WIT (53 IQR: 43–57 vs. 36 min. IQR: 30–44; *p* < 0.009).

**Conclusion:**

Increased WIT increases the risk of DGF in pediatric kidney transplant recipients, whereas MP appears to be protective against DGF and may ameliorate the adverse effect of CIT. DGF was rare in the cohort and further studies are necessary due to the low incidence of DGF.

## Graphical abstract

In a single‐center retrospective study of 89 pediatric kidney transplant recipients, warm ischemia time > 43.5 min and deceased donor kidneys were associated with delayed graft function (DGF), while machine perfusion appeared protective (0% vs. 26% DGF). These findings suggest machine perfusion may mitigate DGF risk in pediatric kidney transplantation.

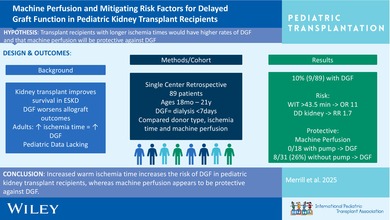

**Abbreviations**
BMI: body mass index
CCHMC: Cincinnati Childrens Hospital Medical Center
CKD: chronic kidney disease
cPRA: calculated panel reactive antibody
DCD: donation after circulatory death
DGF: delayed graft function
ESKD: end‐stage kidney disease
HLA: human leukocyte antigens
IRI: ischemia reperfusion injury
KDPI: kidney donor profile index
MM: mismatches
NAPRTCS: North American Pediatric Renal Trials and Collaborative Studies
REDCap: research electronic data capture
RR: relative risk

## 1 Introduction

Kidney transplantation is the preferred kidney replacement modality for children diagnosed with end‐stage kidney disease (ESKD) and late‐stage chronic kidney disease (CKD), ideally before dialysis is initiated [1, 2]. Children and adolescents who receive kidney transplants from living donors (LD) have better 1‐ and 5‐year allograft survival rates compared to those who receive deceased‐donor (DD) kidneys [3]. These differences may be due, in part, to the intensive screening of living donors compared to the ill effects of brain death that induces a pro‐inflammatory state or traumatic brain death which may induce complement activation and thrombotic microangiopathy in deceased donors [4, 5]. Another important factor is limited ischemia time because living donor nephrectomy is timed to coordinate with the recipient transplant [4].

Ischemia–reperfusion injury (IRI) is a known mechanism of injury in kidney transplantation. IRI occurs in both deceased donor and living donor kidney transplants, although ischemia times are longer in deceased donor transplants [6]. Delayed graft function (DGF) is thought to be primarily a consequence of IRI causing acute tubular necrosis [7]. Each hour of cold ischemia time increases the risk of DGF in adult patients [8], but this is not as well studied in the pediatric population. A study from the North American Pediatric Renal Trials and Collaborative Studies (NAPRTCS) registry showed prolonged cold ischemia time (over 24 h) was associated with higher rates of DGF [9]. Bousetta et al. noted that cold ischemia times (over 20 h) were associated with DGF in pediatric patients, but did not see any difference in warm ischemia time looking at a cutoff of 40 min [10]. One other study noted that cold ischemia time over 12 h was associated with DGF [11] in patients over 12 years old. However, other than analysis of these crude cut‐offs, there are limited data linking ischemia times to outcomes in children. Even though children are usually receiving adult organs, it is important to study risk and protective factors in children specifically, primarily because there are significant hemodynamic differences between adult and pediatric kidney transplant recipients. To ensure adequate perfusion of the adult donor kidney, pediatric kidney transplant recipients may require more liberal use of fluids and vasoactive support which may impact the effects of ischemia time and other factors on DGF [12, 13].

Increasingly, organ procurement organizations are utilizing machine perfusion during transport of kidney allografts which is associated with a reduced risk DGF in adult patients [14, 15]. There are no published data evaluating the outcomes of machine perfusion in pediatric kidney transplant recipients. In this retrospective analysis, we sought to evaluate risk factors for DGF, including donor organ type, warm and cold ischemia time, and machine perfusion in our single center. We hypothesized that transplant recipients with longer ischemia times would have higher rates of DGF and that machine perfusion will be protective against DGF.

## 2 Methods

This was a retrospective cohort analysis of patients who received a kidney transplant at Cincinnati Children's Hospital Medical Center (CCHMC) from 11/1/2017 to 11/30/2021. The study was approved by the Institutional Review Board (IRB) at CCHMC. Patients aged 18 months to 21 years old during the defined period were included. Patients were excluded if they received a multi‐organ transplant or had recurrence of primary disease as the indication for kidney replacement therapy post‐transplant. There was a simultaneous prospective study of the same cohort and thus, if patients did not consent to the prospective study, they were not included in this cohort as they did not consent to data collection per the IRB guidelines. Data were obtained from the hospital electronic health record or patient's UNOS transplant file by automated data extraction or manual extraction. Study data were collected and managed using Research Electronic Data Capture (REDCap) capture tools hosted at CCHMC [16, 17].

### 2.1 Statistical Analysis

Continuous descriptive data were reported using mean and standard deviation for normally distributed data or median with interquartile range for non‐normally distributed data. Categorical data were reported as number of the cohort and proportion percentage. Continuous predictor data were analyzed using Wilcoxon rank‐sum test as all data were non‐normally distributed determined by use of the Shapiro–Wilk test. Categorical predictor data were analyzed via Fischer's exact test. Relative risk (RR) was calculated directly for categorical predictors. Univariate RR with 95% confidence interval (CI) was estimated for each predictor using Poisson regression with a log link and robust (sandwich/Huber‐White) variance estimation to account for the non‐Poisson‐distributed nature of the binary outcome. This approach was chosen over log‐binomial regression due to more consistent model convergence, particularly given sparse event counts in some strata. In the event Poisson regression was unstable, models were fit using a bias‐reduced (Firth‐type) estimation approach, implemented via the mixed adjusted score equations method. All analyzes were performed separately within each donor‐type subgroup (LD, DD‐MP, DD‐non‐MP) using R (version [4.6.1]; R Core Team) with the stats, sandwich, and lmtest packages.

To determine optimal cutoff of warm ischemia time to the outcome of DGF, a Youden's index was calculated.

The primary outcome (dependent variable) was the presence of DGF defined as the need for dialysis (hemodialysis, peritoneal dialysis, or continuous kidney replacement therapy) during the first 7 days post‐transplant. Dialysis need was determined by the treating physician on the inpatient service at the time of the recipient's care. Baseline characteristics were compared between the living donor (LD), deceased donor machine perfusion (DD‐MP), and deceased donor non‐machine perfusion (MP) recipients and predictors of DGF were analyzed in the entire cohort, then in the separate sub‐categories. Predictors (independent variables) of interest included patient biological sex, race, patient age, patient body mass index (BMI), etiology of kidney disease, prior kidney transplant, pretransplant dialysis, pretransplant calculated panel reactive antibody (cPRA), donor type (living or deceased), human leukocyte antigen (HLA) mismatch, ischemia time (warm, cold, total) and for deceased donors, kidney donor profile index (KDPI), DBD or DCD for deceased donors, and presence or absence of machine perfusion. Beyond KDPI, DBD, and DCD other donor characteristics were not available for analysis. Warm ischemia time was defined as the time from anastomosis to unclamping along with any additional ischemia time off ice as noted by the transplant surgeon while in the operative field. The warm ischemia time does not include any donor “warm time” during DCD procurement. With only 9 DGF events observed among 89 patients, this cohort does not meet the minimum events‐per‐variable threshold (typically 10–15 events per predictor) required to support a stable multivariable logistic regression model. Attempts to incorporate multiple predictors resulted in unstable effect estimates, reflected by extremely wide confidence intervals (e.g., spanning several orders of magnitude for some donor‐type categories), indicating sparse data within specific subgroups rather than genuine, generalizable associations. Given this constraint, multivariable modeling was not pursued as a primary analysis; instead, associations between individual predictors and DGF were assessed independently to preserve statistical validity and avoid overfitting. Significance level was set at an alpha of 0.05. All comparison tests performed were two‐sided tests. Statistical analysis was completed using RStudio (version [4.6.1]; R Core Team).

## 3 Results

**FIGURE 1:**
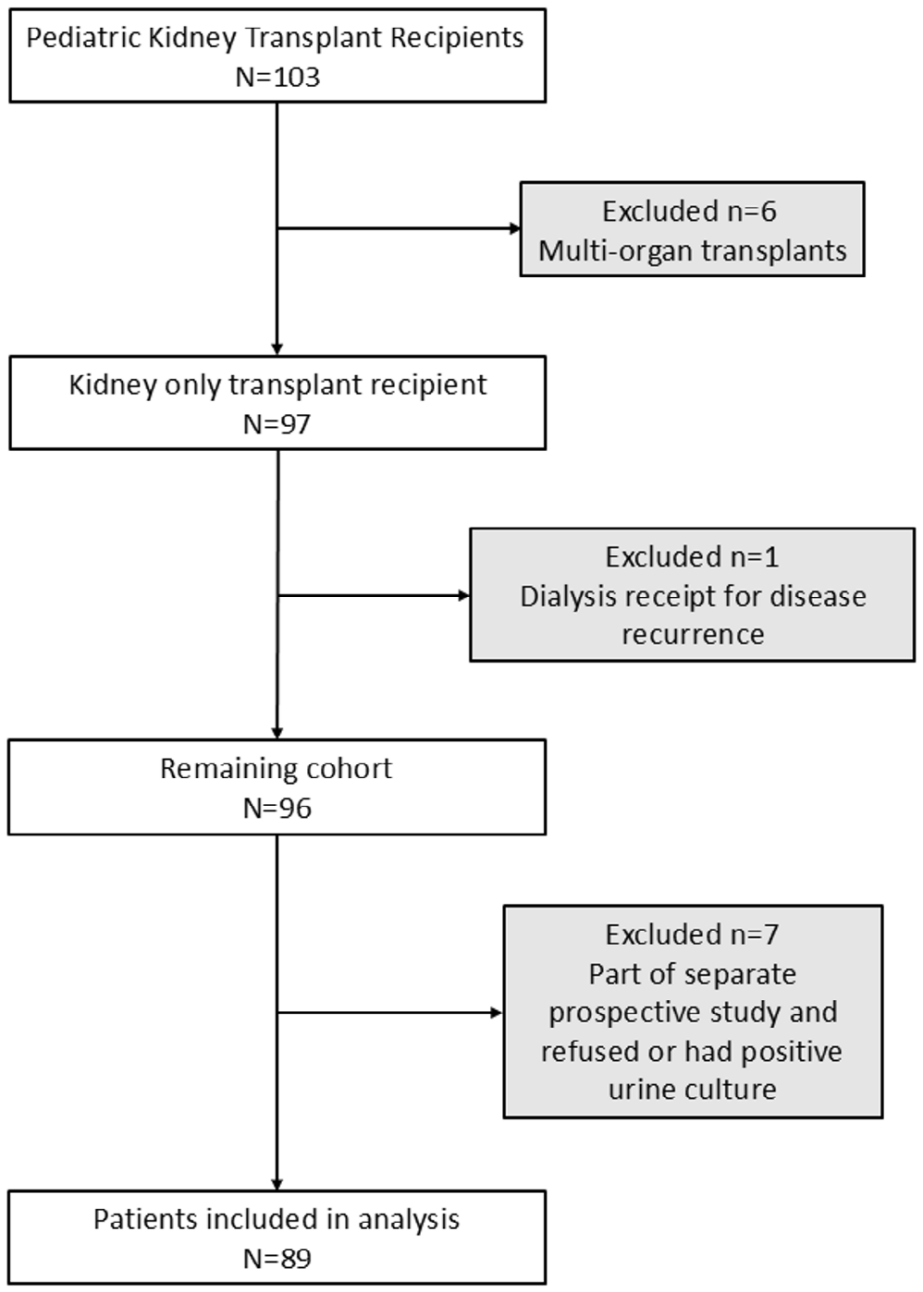
Flow Consort Diagram of Kidney Transplant Recipients during the Study Timeframe.

103 patients received a kidney transplant during the study period. 14 patients were excluded due to receiving multi‐organ transplantation, receipt of dialysis for disease recurrence, or being excluded for a prospective study of the same patient population (Figure 1) leaving 89 subjects included in the analysis.

Of these 89 patients, 49 (55%) received a deceased donor kidney transplant with the remaining 40 (45%) receiving a living donor transplant. Table 1 shows a comparison of baseline characteristics of the total cohort, living donor recipients and deceased donor recipients where deceased donor recipients are further split into receipt of machine perfusion or no machine perfusion allografts. Overall, the cohort was more than half male (57%) and predominantly Caucasian (88%). Thirty‐five percent had primary glomerular disease and most (75%) were on dialysis prior to transplant. Deceased donors of both types were older and more likely to be African American. There was no difference in proportion of deceased donor recipients from donors after circulatory death between the groups. Total HLA mismatch varied significantly across donor types as well as ischemia times, in both cold and total ischemia times. Deceased donor kidney recipients, both machine and non‐machine perfusion recipients were more likely to receive t‐cell depleting induction immunosuppression.

**TABLE 1:** Baseline characteristics of the cohort including comparison of living donor versus deceased donor machine perfusion kidney versus deceased donor non‐machine perfusion kidney transplant recipients.

	Total cohort (*n* = 89)	Living donor recipients (*N* = 40)	Deceased donor—machine perfusion (*n* = 18)	Deceased donor—no machine perfusion (*N* = 31)	*p* ^b^
**Demographics**					
Male	51 (57%)	25 (63%)	8 (44%)	18 (58%)	0.46^c^
African American	11 (12%)	2 (5%)	5 (28%)	4 (13%)	0.05^c^
Age at transplant (y)^a^	11.9 (4.9–16.4)	10.1 (4.1–14.6)	12.2 (3.1–16.6)	14.8 (9.6–17.4)	0.03^d^
BMI at transplant^a^	17.6 (16.4–20.6)	17.3 (16.2–19)	17.7 (16.1–21.0)	19.1 (16.9–22.1)	0.21^d^
**Primary glomerular diagnosis**	31 (35%)	13 (33%)	3 (17%)	15 (48%)	0.08^c^
**Prior kidney transplant**	6 (7%)	1 (2%)	2 (11%)	3 (10%)	0.34^c^
**Pretransplant Dialysis**	67 (75%)	30 (75%)	11 (61%)	26 (84%)	0.19^c^
**Pretransplant cPRA** (%)^a^	0 (0–1)	0 (0–0)	0 (0–19)	0 (0–1.5)	0.68^d^
**Deceased donation after cardiac death**	12 (24%)	—	4 (22%)	8 (26%)	> 0.9
**KDPI**	13 (5–20)		12 (7–20)	14 (3–21)	> 0.9
**HLA mismatch**					
Total^a^	3 (2.3–5)	3 (2–4.3)	3 (3–4)	4 (3–5)	0.04^d^
0 MM	1 (1%)	1 (2%)	0 (0%)	0 (0%)	1^d^
1–5 MM	86 (97%)	37 (93%)	18 (100%)	31 (100%)	
6 MM	2 (2%)	2 (5%)	0 (0%)	0 (0%)	
**Ischemia time (min)** ^**a**^					
Cold ischemia	408 (43–787)	41 (35–54)	929 (862–958)	571 (331–713)	< 0.001^d^
Warm ischemia	38 (32–45)	36 (29–43)	39 (36–42)	39 (32–49)	0.51^d^
Total ischemia	344 (82–826)	80 (71–98)	966 (899–1010)	601 (370–775)	< 0.001^d^
**T‐cell depleting induction**	16 (18%)	3 (8%)	4 (22%)	9 (29%)	0.05^c^

^a^ Median (IQR).

^b^ comparing LD to DD‐MP to DD‐NoMP.

^c^ Fisher's exact test.

^d^ Kruskal–Wallis test.

Nine of 89 (10%) patients developed DGF (Table 2). Donor organ type (LD vs. DD‐MP vs. DD‐non‐MP) was associated with development of DGF (*p* = 0.02). When comparing both LD and DD‐MP to the reference group of DD‐non‐MP, DD‐MP trended toward less DGF but did not reach statistical significance. However, LD had a RR of 0.14 (95% CI 0.026–0.74, *p* = 0.02) and was associated with a lower likelihood of developing DGF compared to DD‐non‐MP in this cohort. Patients who developed DGF had longer warm ischemia times (49 min, IQR: 44–57 vs. 37 min, IQR: 30–43; *p* = 0.002) but there was no difference in cold or total ischemia times. The lowest warm ischemia time that resulted in DGF was 35 min. Youden's index calculation estimated an optimal cutoff for warm ischemia time as a predictor of DGF to be 43.5 min (sensitivity 78%, specificity 76%, AUC 0.80). Patients with warm ischemia time greater than 43.5 min were 11‐fold more likely to experience DGF than those with warm ischemia time less than 43.5 min (OR 11.2; 95% CI 2.5–80, *p* = 0.004). To assess whether risk continued to increase progressively with longer warm ischemia time above this threshold, we evaluated the odds of DGF per additional 5‐min increment among patients with warm ischemia time ≥ 43.5 min (*n* = 26, 7 events). No significant association was observed (OR 1.00, 95% CI 0.80–1.07, *p* = 0.92).

**TABLE 2:** Analysis of predictors of delayed graft function in all donor types.

	Delayed graft function (*n* = 9)	No delayed graft function (*n* = 80)	Univariate *p*	Relative risk (95% CI)
**Demographics**				
Male	4 (44%)	47 (59%)	*0.49* ^b^	0.8 (0.4–1.6)
African American	2 (22%)	9 (11%)	*0.31* ^b^	2.0 (0.5–7.8)
Age at transplant (y)^a^	13.8 (11.6–17.7)	11.5 (4.1–16.3)	*0.12* ^c^	1.1 (0.98–1.3)
BMI at transplant^a^	22.9 (16.9–31.6)	17.6 (16.3–19.9)	*0.09* ^c^	1.2 (1.0–1.3)
**Primary glomerular diagnosis**	5 (56%)	26 (33%)	*0.27* ^b^	1.8 (0.9–3.3)
**Prior kidney transplant**	0 (0%)	6 (8%)	*1* ^b^	0.6 (0.04–10)
**Pretransplant dialysis**	8 (89%)	59 (74%)	*0.44* ^b^	1.2 (0.9–1.6)
**Pretransplant cPRA** (%)^a^	0 (0–0)	0 (0–1.3)	*0.58* ^c^	0.96 (0.83–1.0)
**Donor type**			*0.002* ^b^	
Living donor	1 (11%)	39 (49%)	*0.02* ^d^	0.14 (0.026–0.74)^d^
Deceased donor—machine perfusion	0 (0%)	18 (23%)	*0.11* ^d^	0.10 (0.006–1.70)^d^
Deceased donor—no machine perfusion	8 (89%)	31 (35%)	—	
**Deceased donor – donation after cardiac death**	2 (25%)	10 (24%)	*0.98*	1.03 (0.1–5.4)
**KDPI (%)**	20 (9–27)	11 (5–20)	*0.14*	1.06 (1.00–1.11)
**HLA mismatch**				
Total^a^	4 (4–5)	3 (2–5)	*0.23* ^c^	1.4 (0.8–2.5)
0 MM	0 (0%)	1 (1%)	*1* ^b^	1.0 (1.0–1.1)^e^
1–5 MM	9 (100%)	77 (96%)		
6 MM	0 (0%)	2 (3%)		
**Ischemia time (min)** ^**a**^				
Cold ischemia	464 (329–788)	271 (41.3–788)	*0.16* ^c^	1.0 (1.0–1.0)
Warm ischemia	49 (44–57)	37 (30–43)	*0.002* ^c^	1.1 (1.0–1.1)
Total ischemia	520 (378–811)	306 (80.3–827)	*0.14* ^c^	1.1 (1.0–1.1)
**T‐cell depleting induction**	2 (22%)	14 (18%)	*0.66* ^b^	1.3 (0.3–4.7)

^a^ Median (IQR).

^b^ Fisher's exact test.

^c^ Wilcoxon rank‐sum test.

^d^ Comparing to DD‐Non‐MP as reference.

^e^ RR comparing 1–5 MM to combination of 0 and 6 MM.

**FIGURE 2:**
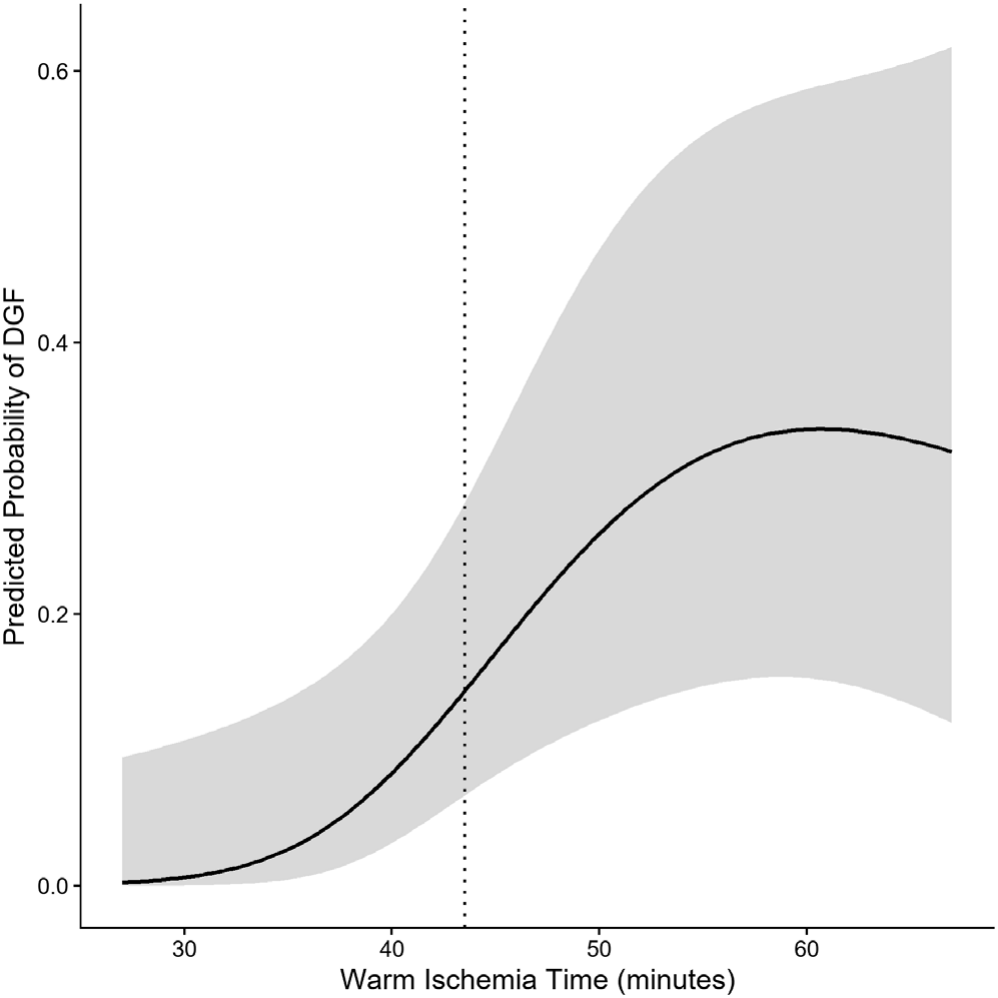
Restricted cubic spline model showing the predicted probability of delayed graft function (DGF) across the observed range of warm ischemia time. The solid line represents the model‐predicted probability of DGF, and the shaded region represents the 95% confidence interval. The dotted vertical line indicates the optimal cutoff (43.5 min) identified via receiver operating characteristic curve analysis using the Youden index.

To further characterize the shape of this relationship across the full range of warm ischemia time, we modeled warm ischemia time as a continuous, non‐linear predictor of DGF using restricted cubic splines (3 knots) in a logistic regression framework (Figure 2). Predicted probability of DGF rose from 0.6% at 30 min to 14.3% at the 43.5‐min cutoff, continuing to rise to a peak of approximately 33.6% around 60 min, after which the curve plateaued. The steepest rise in predicted risk occurred between approximately 40 and 55 min, with the identified cutoff (43.5 min) marking the onset of this high‐risk region rather than its peak. Confidence intervals widened substantially at the upper range of warm ischemia time, reflecting fewer observations beyond approximately 50–55 min.

Next, each donor type subset (living donors, deceased donor‐machine perfusion, and deceased donor non‐machine perfusion) was evaluated to look for predictors of DGF (Table 3). In the living donor cohort, only 1 patient developed DGF, and no predictors reached statistical significance. Interestingly, in the deceased donor‐machine perfusion cohort, 18 patients were included and none of the 18 patients developed delayed graft function. Lastly, in evaluating patients who were recipients of deceased donor non‐machine perfusion allografts, there was no significant difference in recipient demographics, diagnoses, immunologic risks or KDPI. The only significant difference was warm ischemia time 53 (IQR 43–57) vs. 36 min (IQR 30–44, *p* = 0.009) with a relative risk of 1.05 (95% CI 1.02–1.08, *p* = 0.001). There was no difference in DCD between DD‐MP (4/18, 22%) and DD‐non‐MP (8/31, 26%) donors occurred in 22% of DD‐MP recipients compared to 25% (2/8) of DD‐non‐MP recipients with DGF and 26% (6/23) of DD‐non‐MP without DGF recipients. There were no overall differences in the demographics or other predictor variables when comparing deceased donor machine perfusion versus non‐machine perfusion predictors.

**TABLE 3:** Analysis of patients with and without delayed graft function (DGF) by donor type.

	Living donor transplant			Deceased donor transplant (machine perfusion) *N* = 18		Deceased donor transplant (non‐machine perfusion) *N* = 31				
	DGF (*n* = 1)	No DGF (*n* = 39)	*p*	DGF (*N* = 0)	No DGF (*N* = 18)	DGF (*N* = 8)	No DGF (*N* = 23)	Univariate *p*	Relative risk (95% CI)	RR *p* (95% CI)
**Demographics**										
Male	1 (100%)	24 (62%)	*1* ^*b*^	—	8 (44%)	3 (38%)	15 (65%)	0.23^b^	0.43 (0.13–1.50)	0.19
African American	0 (0%)	2 (5%)	*1* ^*b*^	—	5 (28%)	2 (25%)	2 (9%)	0.27^b^	2.25 (0.67–7.53)	0.19
Age at transplant (y)^a^	18	10.0 (4–14.2)	*0.1* ^c^	—	12.2 (3.14–16.6)	14.3 (11.0–16.5)	14.8 (9.8–17.4)	0.98^c^	1.10 (0.91–1.12)	0.86
BMI at transplant^a^	33	17.2 (16.2–18.9)	*0.1* ^c^	—	17.7 (16.1–21.0)	21.6 (16.7–26.2)	18.7 (17.0–21.0)	0.49^c^	1.07 (1.00–1.14)	0.053
**Primary glomerular diagnosis**	1 (100%)	12 (31%)	*0.33* ^b^	—	3 (17%)	4 (50%)	11 (48%)	*1* ^b^	1.07 (0.32–3.52)	0.92
**Prior kidney transplant**	0 (0%)	1 (3%)	*1* ^b^	—	2 (11%)	0 (0%)	3 (13%)	0.55^b^	0 (0–0)	n/a
**Pretransplant dialysis**	1 (100%)	29 (74%)	*1* ^b^	—	11 (61%)	7 (88%)	19 (83%)	1^b^	1.35 (0.21–8.68)	0.75
**Pretransplant cPRA** (%)^a^	17	0 (0–0)	*0.12* ^c^	—	0 (0–19)	0 (0–0)	0 (0–35)	0.18^c^	0.87 (0.71–1.05)	0.15
**Deceased donor organ**										
Donation after cardiac death				—	4 (22%)	2 (25%)	6 (26%)	1^b^	0.96 (0.24–3.82)	0.95
KDPI (%)^a^				—	11.5 (7.8–20.0)	20 (10.8–26)	10 (3–19)	0.14^c^	1.04 (0.99–1.09)	0.08
**HLA mismatch**										
Total^a^	5	3 (2–4)	*0.23* ^c^	—	3 (3–4)	4 (3.8–4.3)	4 (3–5)	0.45^c^	0.89 (0.54–1.46)	0.63
0 MM	0 (0%)	1 (3%)	*1* ^b^	—	0 (0%)	0 (0%)	0 (0%)	1^b^	—	—
1–5 MM	1 (100%)	36 (92%)		—	18 (100%)	8 (100%)	23 (100%)			
6 MM	0 (0%)	2 (5%)		—	0 (0%)	0 (0%)	0 (0%)			
**Ischemia time (min)** ^**a**^										
Cold ischemia	87	39 (35–51)	*0.14* ^c^	—	929 (862–958)	471 (394–781)	587 (323–679)	< 0.75^c^	1 (0.99–1.00)	0.84
Warm ischemia	48	36 (29–43)	*0.26* ^c^	—	39 (36–42)	53 (43–57)	36 (30–44)	0.009^c^	1.05 (1.02–1.08)	0.001
Total ischemia	135	78 (71–99)	*0.17* ^c^	—	966 (898–1010)	521 (437–852)	626 (353–737)	< 0.71^c^	1 (0.99–1.00)	0.74
**T‐cell depleting induction**	1 (100%)	2 (5%)	*0.08* ^b^	—	4 (22%)	1 (13%)	8 (35%)	0.38^b^	0.35 (0.05–2.45)	0.29

^a^ Median, IQR.

^b^ Fishers exact test.

^c^ Wilcoxan rank‐sum test.

## 4 Discussion

In this cohort, the incidence of DGF occurred in 9 of the 89 patients (10%)—similar to the 12% rate reported by Tejani et al. in their study of over 5000 patients in the NAPRTCS cohort [9] as well as a study by Boussetta et al. that revealed an incidence of DGF in 15% of their cohort of 113 transplant patients [10]. Both Tejani and Boussetta reported a higher incidence of DGF in patients who received deceased donor vs. living donor kidney allografts. Unlike these studies, we found that longer warm ischemia time is associated with DGF, in addition to receipt of a deceased donor allograft. There was no DGF in any patient with warm ischemia time < 35 min, and patients with more than 43.5 min warm ischemia time had roughly 11 times higher odds of DGF. In the deceased donor cohort, warm ischemia time was again found to be predictive of DGF. Importantly, none of the 18 patients who received a deceased donor organ and machine perfusion developed DGF compared to 8/31 (26%) who developed DGF without receipt of machine perfusion. We also found no relationship between CIT and DGF, and observation related to the fact that the highest CITs were observed in the DD‐MP cohort, but no DGF occurred in that cohort. Interestingly, when comparing the 2 cohorts LD and DD‐MP to DD‐non‐MP, DD‐MP compared to DD‐non‐MP did not have statistically significantly different rates to DGF, potentially limited by our low incidence of DGF in our cohort or the low overall sample size of our cohort. Thus, in this cohort, machine perfusion appears to be protective of developing DGF and may completely or partially ameliorate the increased risk of DGF related to CIT and deceased donor compared to living donor kidney transplantation.

In adult transplant recipients, the finding that increased cold and warm ischemia time increases the risk of DGF has been previously reported [8, 11, 18, 19]. Marzouk et al. showed that in deceased donor organs, anastomosis time (defined as the time between the end of cold time and perfusion of the donor kidney) was independently associated with DGF in a multivariable regression analysis with an odds ratio of 1.037 per minute. Additionally, an anastomosis time greater than 29 min was associated with a 3.5 times higher risk of DGF [20]. In a multi‐center study of patients who received a deceased donor kidney from donation after circulatory death, Sun et al. observed that prolonged warm ischemia time was predictive of DGF [18]. Both the adult studies and our study suggest that limiting warm ischemia time is important and that outcomes change with each additional minute of warm ischemia time.

In children, the data regarding these associations of ischemia time and DGF are less well established. The need for pediatric‐specific data is highlighted by the marked difference in hemodynamic status between an adult receiving an adult‐sized kidney and a pediatric recipient, who typically has lower blood pressure and cardiac output yet must still accommodate an adult‐sized graft. Often, provision of intravenous fluids and use of vasoactive medications are utilized to ensure adequate renal perfusion, but the excessive fluid also comes with risk of fluid overload and pulmonary edema [12]. Regarding studies in children, Tejani et al. in their analysis of the NAPRTCS registry found that cold ischemia time > 24 h was associated with DGF [9], but this study did not evaluate more granular timeframes of cold ischemia time nor any aspect of warm ischemia time. More recently, Hwang et al. evaluated all pediatric kidney transplants in the UNOS database and found that DGF was more common in deceased donor organs, but that DGF was not correlated with cold ischemia times [21]. Thus, our findings that increased warm ischemia time is associated with DGF and that when machine perfusion is used CIT is not, are important additions to our understand of this phenomenon in children.

Some organ procurement organizations have been utilizing machine perfusion more frequently in the transportation of deceased donor kidneys. A large, randomized, controlled trial by Moers et al. in 2009 randomly assigned one kidney from 336 consecutive donors to machine perfusion and the other donor kidney to static hypothermic perfusion. They found that machine perfusion was associated with an adjusted odds ratio of 0.57 for the risk of development of DGF as well as improved long term allograft survival [15]. Moers et al. also flagged that DCD and expanded criteria donors (ECD) kidneys would have the largest clinical benefit [15]. Treckmann et al. then did a further subgroup analysis looking at ECD‐DBD kidneys showing a benefit in this population [22]. More recently in 2021, Singh et al. found that organs that underwent machine perfusion had better early graft function [14]. There is no published data on the utility of machine perfusion in pediatric kidney transplant recipients, or which donors would benefit the most from the use of machine perfusion. Our study included 49 total patients who received deceased donor kidney transplants. 18 of the 49 (16%) received machine perfusion during transportation to the recipient hospital and none of those 18 (0%) patients developed DGF compared to 8 of 31 patients (26%) who did not receive machine perfusion. DCD donor rates were similar in our cohort across all both DD‐MP and DD‐non‐MP with and without DGF. Our results together with the cited previous studies suggest that machine perfusion should be utilized more often in the transport of deceased donor organs and may decrease or eliminate the increased risk of DGF associated with deceased donor transplant.

Machine perfusion comes with additional costs. A cost‐effectiveness assessment was completed by Gomez et al. for adult patients that showed, despite the additional cost of machine perfusion, there was an overall cost‐savings in the avoidance of DGF and primary non‐function in the short term [23]. Most specifically, Groen et al. showed with a Markov model over 10 years, there was a cost‐savings of $86 750 per quality‐adjusted life‐year in the long term [24]. The cost‐effectiveness for machine perfusion in children has not been performed, but in light of our findings machine perfusion appears to be justified.

We must interpret these results considering several limitations inherent to a single center, retrospective study. Our limited sample included only 9 total patients with DGF and a total of 89 patients, which is likely underpowered to reach statistical significance across several variables with wide heterogeniety. Additionally, this small sample size and number of DGF outcomes limited the ability to perform a robust multivariable analysis. Attempts to incorporate multiple predictors resulted in unstable effect estimates, reflected by extremely wide confidence intervals indicating sparse data within specific subgroups rather than genuine, generalizable associations. Given this constraint, multivariable modeling was not pursued as a primary analysis; instead, associations between individual predictors and DGF were assessed independently to preserve statistical validity and avoid overfitting. Additionally, we found only 1 recipient of a living donor transplants that developed DGF and therefore a specific analysis of living donor recipients could not be performed. In the analysis of warm ischemia time and DGF outcome, confidence intervals widened at the upper range of warm ischemia time, reflecting fewer observations in this range and limiting precision of risk estimates beyond approximately 50–55 min. One should also evaluate the finding of significant difference in HLA mismatch with caution as we found overall difference in total HLA mismatch but noted no difference in our chosen groups, and likely had we chosen different grouping cutoffs, this likely would have resulted in different results and potentially could have determined an optimal mismatch number, but as that was not the intent of this paper, this was not completed. The retrospective nature of the study allowed the inclusion only of data already present in the electronic medical record or UNOS file. Additionally, we acknowledge that we are missing key data points that are risk factors of DGF such as donor age, kidney laterality, and donor cause of death. Additionally, the definition of DGF as dialysis in the first week following transplant has several limitations associated with baseline risk of recipient (e.g., patients who are anuric prior to transplant are more likely to require dialysis if a transplanted kidney is slow to recover than those receiving a pre‐emptive transplant). The observation that the DD‐MP cohort had relatively fewer (but not statistically significant) patients with pre‐transplant dialysis than DD‐non‐MP patients (61% vs. 84%; *p* = 0.19) implies that our result may be biased toward fewer DGF events in this group. There could be other factors that bias this outcome in unpredictable ways such as differences in provider threshold for initiating dialysis, anuric prior to transplant etc. Nonetheless, this is a definition widely used in research of this topic. Finally, data regarding machine perfusion specifics (flow rates and resistances) was not available. This might have led to selection bias; if an organ had high resistances or low flows on pump, it may not have been transplanted, thus underestimating the incidence of DGF by not utilizing an organ that would have developed DGF. Even so, this diagnostic dimension of machine perfusion is a positive feature of this technique and likely helps avoid the acceptance of organs likely to result in DGF [25, 26]. Given these limitations, we believe nonetheless that these data add important information to kidney transplantation in children and stimulate future research and larger studies to find true causal relationships.

## 5 Conclusion

Longer warm ischemia time and receipt of a deceased donor organ were associated with DGF in our single center experience. In those patients who received deceased donor organs, machine perfusion appears to be protective and may mitigate the adverse effects of CIT and/or reduce the risk DGF. Future larger studies should be performed to better understand which risk factors are most important in developing DGF and specifically how much a specific incremental increase in cold or warm time increases this risk in pediatric patients.

## Funding

The authors have nothing to report.

## Disclosure

K.A.M. reports consulting relationship with BioPorto Diagnostics Inc. and is KOL for Vantive, INC. S.L.G. reports consulting income and grant funding from BioPorto Diagnostics Inc., which markets ProNephroAKI. S.L.G. also reports consulting income and grant funding from Calcimedica, Vantive, SeaStar Medical, Talphera, and Otsuka. S.L.G. has additional consulting income from Silver Creek Pharmaceuticals and TRiNDS Inc. S.L.G. is a stockholder of MediBeacon Inc. During conduct and writing of this research, D.K.H. reports consulting income from BioPorto Diagnostics Inc., Alynylam Pharmaceuticals, Kaneka America, and Maximus Federal. D.K.H. currently reports consulting income from AssureCare LLC. D.K.H. was at Cincinnati Children's Hospital and University of Cincinnati at the time this research was conducted. He is now faculty at the University of Utah Spencer Fox Eccles School of Medicine, Salt Lake City, Utah.

## Data Availability

The data that support the findings of this study are available from the corresponding author upon reasonable request.
